# The promotion of functional expected teaching-related emotions through expressive writing

**DOI:** 10.1371/journal.pone.0267905

**Published:** 2022-05-02

**Authors:** Markus Forster, Christof Kuhbandner

**Affiliations:** Department of Human Sciences, University of Regensburg, Regensburg, Germany; Yamaguchi University: Yamaguchi Daigaku, JAPAN

## Abstract

The aim of the present preregistered study was to examine whether expressive writing can help teacher students to develop functional expected teaching-related emotions. In a variation of James W. Pennebaker´s expressive writing paradigm, 129 teacher students were randomly assigned to write on three consecutive days either about the future teaching-related events that personally trigger the greatest fear and joy (treatment group: *n* = 67) or about a walk in a forest and a city park (control group: *n* = 62). In both groups, expected teaching-related positive emotions increased and expected teaching-related negative emotions decreased with increased writing sessions. After the writing sessions, the treatment group reported a stronger change in their view about their future professional life as a teacher, a more active personal involvement with their future professional life, and an increased motivation to use expressive writing in the future. These results demonstrate that expressive writing is a promising tool to promote teacher students’ expected teaching-related emotions.

## Introduction

Developing functional expected teaching-related emotions and deriving which competencies have to be acquired to reach emotional well-being in their future professional life is a core element of teacher training. Therefore, the aim of the study was to evaluate whether expressive writing may be a suitable method to promote teacher students’ development of functional expected teaching-related emotions, to initiate reflections about to-be-acquired emotional competencies, and to induce a more active involvement with their future professional life.

Expressive writing is a method where individuals write about emotional events and express their thoughts and feelings surrounding it. The method has been developed primarily by James W. Pennebaker in the late 1980s [1–4]. In the original version of the expressive writing paradigm, termed the Written Emotional Disclosure paradigm, participants are randomly assigned to one of two groups. In the experimental group, participants are instructed to write about past emotional negative experiences (i.e., traumatic or negative life experiences) and to express their thoughts and feelings surrounding it. In the control group, participants are asked to write about a non-emotional topic (e.g., a particular room). Typically, participants repeatedly write about the respective topics for around 15–20 minutes per writing session over several consecutive days. Research has shown that expressively writing about negative experiences has many benefits across a range of health and non-health outcomes: It can decrease depression and lowering depressive symptoms [5, 6], decrease blood pressure [7] and lead to a reduction in consultations [8] or absenteeism from work [9]. Moreover, the use of the expressive writing in educational fields can improve the physical health of undergraduates [10], increase students’ exam performance [11] and reduce test anxiety [12], and improve teachers’ physical health [13].

Research has also shown that not only writing about negative experiences but also writing about positive experiences can be beneficial for physical and psychological well-being. Expressively writing about positive experiences can enhance positive mood [14], reduce depression and perceived stress reactivity [15], lead to better subjective well-being [16], can promote physical and psychological health benefits [17, 18], and reduce teachers’ anxiety [19]. In particular, there is even some evidence that writing about positive experiences may be more beneficial for overall well-being than writing about negative experiences [20, 21], although there are also contradictory findings [22].

Taken together, expressively writing about emotionally positive and negative experiences seems to be a promising technique to enhance physical and psychological well-being. However, the general effect sizes are difficult to evaluate from the current state of research. Although a large number of existing meta-analyses suggests that both physical and psychological health is improved [23–26], other meta-analyses have failed to support the efficacy of expressive writing [27–31]. Thus, it remains to be explored whether the mixed findings might be due to the number of participants and writing sessions, the specificity of the topic, additional support, or something else. Nevertheless, the largest meta-analyses from Frattaroli [25] showed that expressive writing promotes psychological health, physical health, and overall functioning.

Furthermore, studies such as those by Bain et al. [32] have shown that the writing of journal articles about cognitive and experiential topics can increase teacher students’ reflectivity. Accordingly, there may even be more potential in expressive writing in the context of teacher training in that writing may be helpful to become aware of teaching-related joys or problems one was previously unaware of. Furthermore, expressive writing may help to deal with unsolved problems that trigger unwanted brooding, which hinders to see the positive aspects of teaching [33] and introduces a risk factor for experiencing depressed and anxious moods [34]. Finally, expressive writing may help to clarify necessary personal competencies that should be acquired to enhance the positive side and minimize the negative side of teaching, a hypothesis that is supported by studies showing that mental event simulation can benefit problem-solving [35].

Taken together, the described findings suggest that expressive writing may be a suitable method to improve teacher students’ expected teaching-related emotions, to initiate reflections about to-be-acquired emotional competencies, and to induce a more active involvement with their future professional life. Since, to our knowledge, the use of expressive writing in teacher training has not been examined in previous research, the aim of the present study was to examine the effects of expressive writing in teacher training.

Teacher students were randomly assigned to either an expressive writing group where they wrote on three days in a row ten minutes each about the future teaching-related event they feared most and the future teaching-related event they looked forward to the most, or to a control group where they wrote about a walk in the forest and a city park. We measured the effects of expressive writing on their affective state regarding their future everyday professional life as a teacher, their expected emotions regarding the events they had written about, on changes in their views about their future professional life as a teacher, and on their motivation to use expressive writing in the long run.

We hypothesized that participants in the expressive writing group show an improvement in their overall affective state regarding their future everyday professional life as a teacher (i.e., decreased negative affect and increased positive affect), a decrease in experienced fear regarding the fearful future teaching-related event they had written about, and an increase in experienced joy regarding the joyful future teaching-related event they had written about. In addition, we expected that after the writing exercise, teacher students in the expressive writing group compared to the control group are more motivated to use the writing exercises in the long term, and show are more active personal involvement with their later professional life.

## Method

### Participants

The experiment was preregistered (https://osf.io/8skfa). The preregistered target sample size was 128 participants. Initially, 171 participants completed the writing session on the first day, 150 participants on the second day, and 148 on the third day. As the writing sessions had to be completed on three consecutive days according to our preregistration, a sample of 129 German teacher students (104 women, 24 men, 1 without specification) remained for analysis. The mean age was 21.21 years (ranging from 18 to 31 years, *SD* = 2.30), 45.7% were primary school teacher students and 52.7% were secondary school teachers (lower track schools: 8.5%, intermediate track schools: 9.3%, comprehensive schools: 34.9%; others: 1.6%). Participants were paid 15 Euros for full participation. The study was conducted in accordance with the Helsinki Declaration and the University Research Ethics Standards of the University of Regensburg. All participants provided written informed consent. In Germany, these types of psychological studies do not require ethical approval of an Ethics Committee (see https://www.dfg.de/foerderung/faq/geistes_sozialwissenschaften/).

### Design

A mixed factorial design was used with the within-subjects factor of measurement time (4 levels: before the first writing session, after each of the three writing sessions) and the between-subjects factor of group (expressive writing group versus control group). The assignment of participants to groups was random.

### Material and procedure

**Fig 1:**
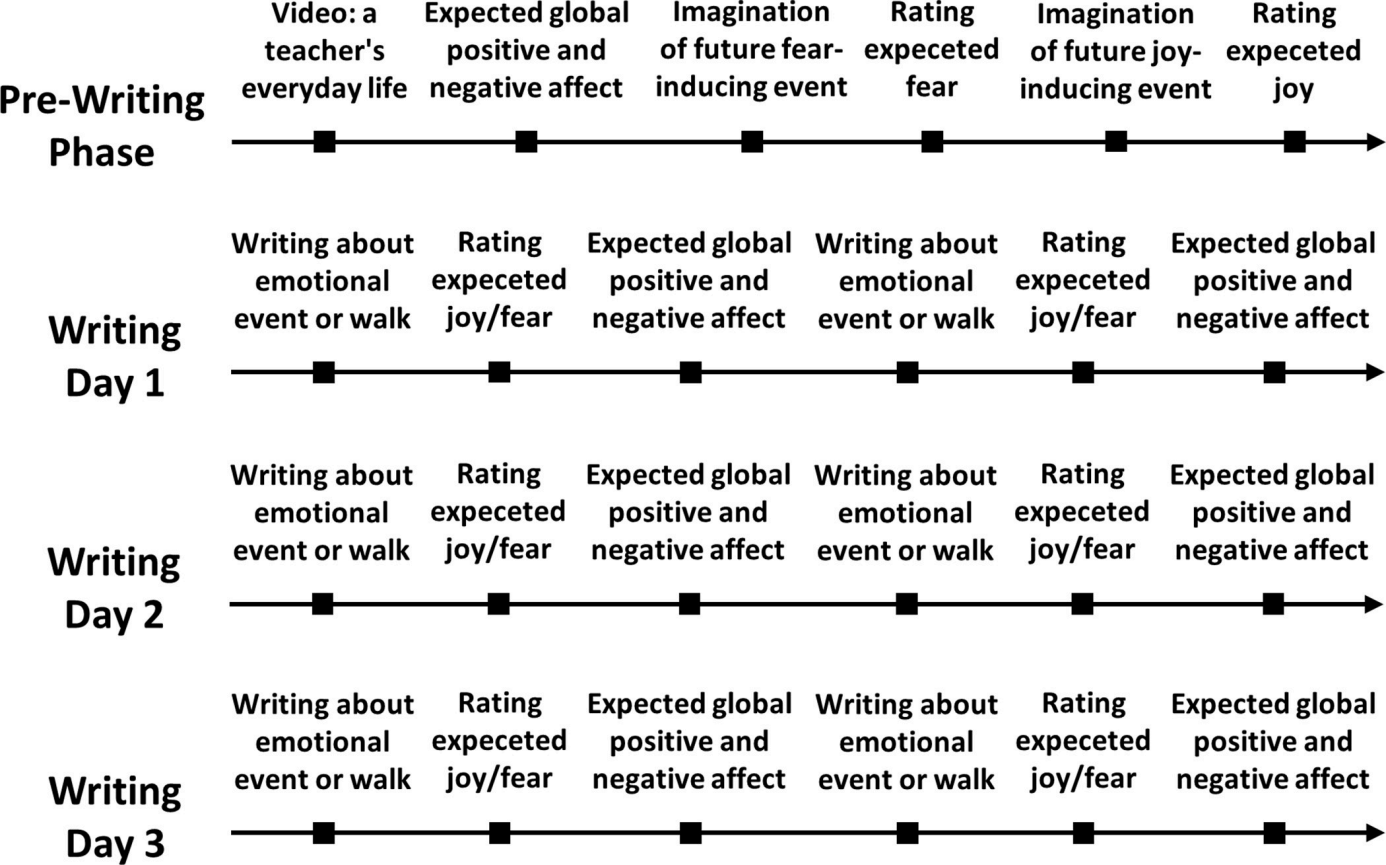
Illustration of the procedure of the study. In an initial pre-writing phase, inspired by a video illustrating a teacher’s everyday working life, participants were asked to rate their expected global positive and negative affect when imagining their future professional life as a teacher, and to think about and emotionally rate the teaching-related events they are most afraid of and most looking forward to. In three subsequent writing phases taking place on three consecutive days, participants were either asked to expressively write about the teaching-related events they are most afraid of and most looking forward to (expressive writing group), or to write about a walk in a forest and a city park (control group). After each of the writing sessions, participants were asked to rate their expected fear and joy associated with the teaching-related event they are most afraid of and most looking forward to, and to rate their expected global positive and negative affect associated with their future professional life as a teacher.

The study was conducted online via SoSci Survey [36]. An overview about the procedure of the study is provided in Fig 1. Both experimental groups wrote on three consecutive days in two writing blocks of 10 min each about two topics. The expressive writing group wrote about the events of their future everyday professional life which personally triggers fear and joy (for details, see below), the control group wrote about a walk in a forest or a city park. In the expressive writing group, half of the participants started with the joy topic and the other half with the fear topic, in the control group, half started with the forest topic and the other half with the city park topic.

In both groups, at the beginning of the experiment, a video about a teacher’s everyday life was shown in order to activate a comprehensive picture of the future professional practice. The video contained 15 different scenes about teaching-related events (e.g., starting a lesson, parent-teacher talk, conflict on the school yard, correcting school work at home) which were illustrated by drawings and accompanied by oral explanations (for details, see https://osf.io/n6e3y/). Next, in both groups, participants were asked to imagine what their future professional life as a teacher will look like, and which emotions they are experiencing when doing so (i.e., expected global positive and negative affect; for details, see below). After that, in both groups, participants were asked to think about which event in their future professional life as a teacher they are most afraid, and which event they are most looking forward to, and to enter the respective events in bullet points in a textbox. After entering the event they are most afraid of, their expected fear was measured, and after entering the event they are most looking forward to, their expected joy was measured (i.e., expected event-related fear and joy; for details see below).

Then, the experimental manipulation took place. In the expressive writing group, participants were asked to write about the both events they had previously entered. After writing about the fear-related event, expected fear was measured again, and after writing about the joy-related event, expected joy was measured again. In the control group, participants were asked to write about a walk in a forest or a city park. The guiding considerations were to choose a task where participants are asked to write about a topic as well. Furthermore, the topic should be unrelated to one’s future professional life as a teacher but still motivating enough to write about it on three days in a row.

After writing about a walk in a forest, respectively after writing about a walk in a city park, half of the participants were asked to assess their expected fear regarding the fear-related event they had previously entered, the other half were asked to assess their expected joy regarding the joy-related event they had previously entered. Additionally, after each individual writing block, expected global positive and negative affect regarding their future professional life as a teacher was measured again.

The writing procedures on the second and third days were identical to the procedure on the first day. The participants were asked to write about the same events they had written on the first day, and after writing, the expected fear and joy and the expected global positive and negative affect regarding their future professional life as a teacher was measured again. Finally, after finishing the final writing session and the emotional ratings on the third day, the participants were asked to assess whether the writing sessions have changed their view about and their involvement with their future professional life as a teacher, and whether they are motivated to use expressive writing in the future (for details, see below).

In terms of teaching the method of expressive writing, we followed the typical instruction used in the expressive writing paradigm [15] and adapted it accordingly. The following instruction was given in the expressive writing group:

*“We would now like to ask you to reflect more deeply about the feelings and sensations you will encounter in your everyday professional life as a teacher. In the following, we would like to ask you to write about your deepest thoughts and feelings in relation to your greatest fear [joy] about your future profession as a teacher. Break away from expectations and explore your deepest emotions and thoughts. Please write about whether this fear [joy] could (1) be useful to you, (2) how you could cope with [promote] the fear [joy] if it occurs in later everyday working life, and (3) what preventive measures you will take to cope with [promote] it in a meaningful way during the course of your studies. Do not worry about spelling, sentence structure, grammar, or style.”*

In the control group, the following instruction was given:

*“We investigate the relationship between the written description of an environment and the expected emotions that could arise in future everyday professional life as a teacher. Please write about a walk in the city park [forest] and describe what you see, hear, feel and/or touch there. Please describe as many details as possible and as objectively as possible. Do not worry about spelling, sentence structure, grammar, or style.”*

### Measures

#### Expected global positive and negative affect

The global positive and negative affect experienced when imagining their future professional life as a teacher was measured with the GESIS Panel [37], the German version of the Positive and Negative Affect Schedule PANAS [38], which consists of 20 items describing different feelings and emotions (ten positive items, e.g., “enthusiastic”, and ten negative items, e.g., “distressed”). The participants were asked to assess to what extent they feel the respective emotions when imagining their future professional life as a teacher on a five point Likert scale ranging from 1 (not at all) to 5 (extremely). To measure positive and negative affect, the means across the ten positive and negative items were calculated. In the present sample, reliability on the ten positive and negative items was high (Cronbach’s alphas = .92/.85).

#### Expected event-related fear and joy

The expected fear and joy when imagining the event in their future professional life one is most afraid of, respectively most looking forward to, was measured at four different levels of emotional functioning. Emotional intensity was measured with the item “How intense is your expected fear [joy] when imagining the fear-inducing [joy-inducing] event?”, controllability of the emotional event was measured with the item “To what extent will you be able to control the occurrence of the fear-inducing [joy-inducing] event in your future everyday professional life?”, the expected emotional burden (fear-inducing event), respectively the expected positive motivational activation (joy-inducing event), was measured with the item “How much will the fear-inducing [joy-inducing] event burden you [positively motivate you] in your future professional life?”, and the role the expected emotion induced by the imagined event will play in future professional life in comparison with all other expected future emotions was measured with the item “Compared to all other emotions you will experience later in the course of your job, how much space will the fear [joy] induced by the event take in your future everyday professional life”. The first three items were rated on seven point Likert scales ranging from 1 (very low) to 7 (very high), the fourth item on a ten point percent scale ranging from 1 (10%) to 10 (100%).

#### Motivation to use expressive writing in the future

The motivation to use expressive writing in the future was measured with a scale consisting of the following four items, rated on five point Likert scales ranging from 1 (no) to 5 (yes): “Do you think expressive writing makes sense?”; “Did you find expressive writing helpful?”; “Can you imagine using expressive writing outside the study?”; “Can you imagine using expressive writing in the long term”?. In the present sample, reliability was high (Cronbach’s alpha = .90).

#### Change in the view about the future professional life

The change in the view about one’s future professional life was measured with a scale consisting of the following seven items, rated on five point Likert scales ranging from 1 (no) to 5 (yes): “Do you have the impression that something has changed in you as a result of the writing sessions?”; “Did the writing sessions help you to develop different views about your future professional life as a teacher with regard to fear-inducing events?”; “Did the writing sessions help you develop different views about your future professional life as a teacher with regard to joy-inducing events?”; “Did the writing exercises make you think more about your future professional life as a teacher?”; “Do you now, after the writing sessions, think differently about your future professional life as a teacher?”; “Do you now, after the writing sessions, feel different about your future everyday working life as a teacher?”; “Did the writing exercises motivate you to prepare yourself more actively for your future professional life as a teacher (e.g., selecting certain courses, etc.)?”. In the present sample, reliability was high (Cronbach’s alpha = .86).

### Statistical analyses

To examine the effects of expressive writing, separate analyses of variances (ANOVAs) with the within-subjects factor of measurement point (before the first writing session, after each writing session on days one, two, and three) and the between-subjects factor of group (expressive writing group, control group) were conducted.

## Results

### Expected global positive and negative affect

**Fig 2:**
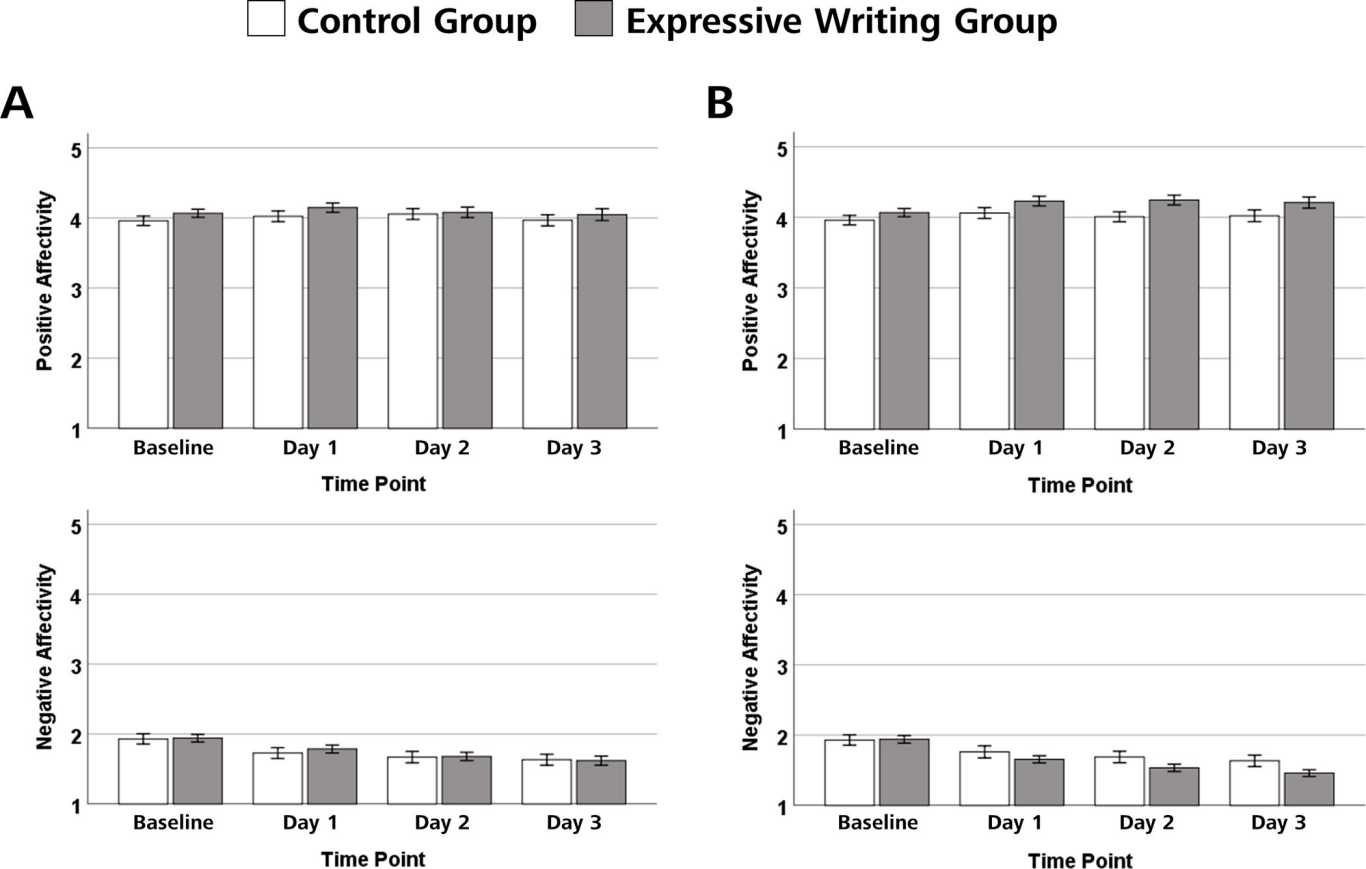
Effects of expressive writing on expected positive and negative affect regarding future professional life as a teacher. The panels in (A) show the expected positive and negative affect after writing about the future teaching-related event one is most afraid of, the panels in (B) show the expected positive and negative affect after writing about the future teaching-related event one is most looking forward to. The heights of the bars show mean positive and negative affect as a function of group (expressive writing group vs. control group) and measurement point (baseline vs. day 1 vs. day 2 vs. day 3). Error bars represent standard errors.

The course of expected positive and negative affect experienced when imagining one’s future professional life as a teacher across writing sessions as a function of experimental group is shown in Fig 2.

Comparing the effects of writing about the fear-inducing event and writing about a walk in the city park/forest revealed a weak and marginally significant main effect of measurement point for positive affect, Greenhouse–Geisser *F*(2.59, 329.24) = 2.51, *p* = .067, *η*_*p*_*^2^* = .019, and a strong significant main effect for negative affect, Greenhouse–Geisser *F*(2.18, 276.96) = 49.38, *ε* = 0.727, *p* < .001, *η*_*p*_*^2^* = .280. Both for positive and for negative affect, there were neither main effects of group, Greenhouse–Geisser *F*(1, 127) = 0.79, *p* = .375, *η*_*p*_*^2^* = .006, and Greenhouse–Geisser *F*(2.59, 329.24) = 2.51, *p* = .067, *η*_*p*_*^2^* = .019, nor interactions between measurement point and group, Greenhouse–Geisser *F*(2.59, 329.24) = 0.77, *p* = .496, *η*_*p*_*^2^* = .006, and Greenhouse–Geisser *F*(2.18, 276.96) = 0.59, *p* = .570, *η*_*p*_*^2^* = .005. Trend analyses showed that for negative affect, there was a linear trend, *F*(1, 127) = 74.5, *p* < .001, *η*_*p*_*^2^* = .370, and a quadratic trend *F*(1, 127) = 16,18, *p* < .001, *η*_*p*_*^2^* = .113, indicating that negative affect decreased with an increased number of writing sessions.

Comparing the effects of writing about the joy-inducing event and writing about a walk in the city park/forest revealed a significant main effects of measurement point for positive affect, Greenhouse–Geisser *F*(2.44, 309.54) = 7.57, *p* < .001, *η*_*p*_*^2^* = .056, and neither a main effect of group, Greenhouse-Geisser *F*(1, 127) = 3.44, *p* = .066, *η*_*p*_*^2^* = .026, nor an interaction between measurement point and group, Greenhouse-Geisser *F*(2.44, 309.54) = 1.54, *p* = .212, *η*_*p*_*^2^* = .012. Trend analyses showed that there was a linear trend, *F*(1, 127) = 5.43, *p* = .021, *η*_*p*_*^2^* = .041, a quadratic trend, *F*(1, 127) = 14.04, *p* < .001, *η*_*p*_*^2^* = .100, and a cubic trend for positive affect, *F*(1, 127) = 4.86, *p* = .029, *η*_*p*_*^2^* = .037, indicating that positive affect increased with an increased number of writing sessions.

For negative affect, there was a main effect of measurement point, Greenhouse–Geisser *F*(2.37, 300.99) = 68.95, *p* < .001, *η*_*p*_*^2^* = .352, no main effect of group, Greenhouse-Geisser *F*(1, 127) = 1.49, *p* = .225, *η*_*p*_*^2^* = .012, and a significant interaction between measurement point and group, Greenhouse-Geisser *F*(2.37, 300.99) = 4.04, *p* = .013, *η*_*p*_*^2^* = .031. For both the expressive writing group and the control group, trend analyses showed linear trends, *F*(1, 66) = 98.55, *p* < .001, *η*_*p*_*^2^* = .599, and *F*(1, 61) = 29.32, *p* < .001, *η*_*p*_*^2^* = .325, and quadratic trends, *F*(1, 66) = 17.39, *p* < .001, *η*_*p*_*^2^* = .209, and *F*(1, 61) = 5.09, *p* = .028, *η*_*p*_*^2^* = .077, indicating that negative affect decreased with an increased number of writing sessions in both groups. As indicated by the effect sizes, the decrease in negative affect was larger in the expressive writing group than the control group.

### Expected event-related fear and joy

**Fig 3:**
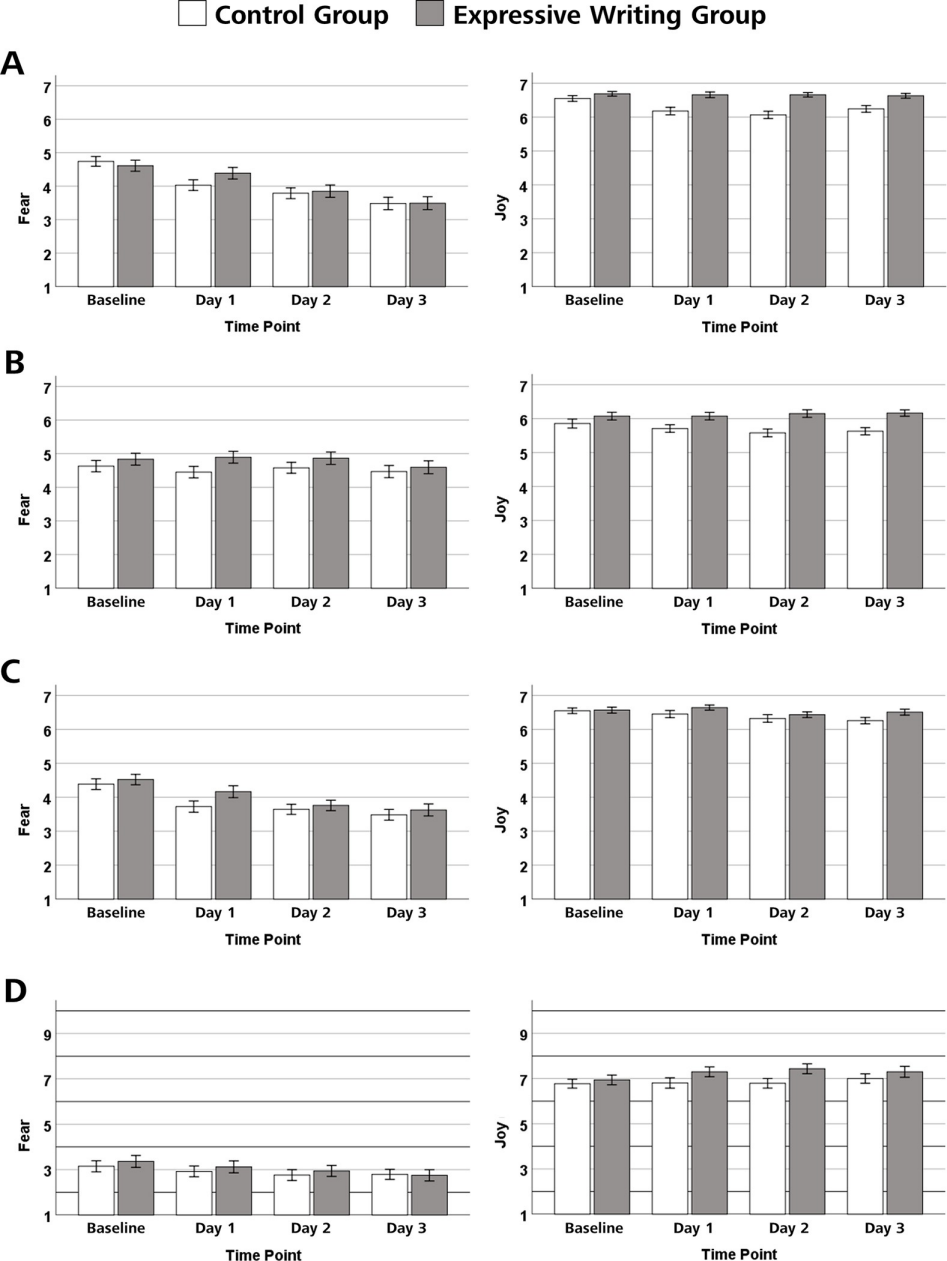
Effects of expressive writing on expected fear and joy elicited by the events one is writing about. The panels in (A) show the intensity of the expected fear and joy, the panels in (B) show the expected controllability of the fear-inducing and joy-inducing events, the panels in (C) show the expected emotional burden (fear) and the expected positive motivational activation (joy), and the panels in (D) show the expected role the elicited emotions will play in future professional life in comparison with other expected emotions. The heights of the bars show the respective means as a function of group (expressive writing group vs. control group) and measurement point (baseline vs. day 1 vs. day 2 vs. day 3). Error bars represent standard errors.

The course of expected fear and joy elicited by the event in future professional life one is most afraid of, respectively most looking forward to, across writing sessions as a function of experimental group is shown in Fig 3. Fig 3A shows the courses for expected intensity, Fig 3B the courses for expected controllability, Fig 3C the courses for expected emotional burden (fear) and expected positive motivational activation (joy), and Fig 3D the courses for the expected role the elicited emotions will play in in future professional life comparison with other expected emotions.

#### Emotional intensity

For expected fear, there was a significant main effects of time, Greenhouse–Geisser *F*(2.41, 305.4) = 43.47, *p* < .001, *η*_*p*_^2^ = .255, no significant main effect of group, Greenhouse–Geisser *F*(1, 127) = .13, *p* = .718, *η*_*p*_^2^ = .001, and no significant interaction between time and group, Greenhouse–Geisser *F*(2.41, 305,4) = 1.73, *p* = .171, *η*_*p*_^2^ = .013. Trend analyses showed that there was a linear trend *F*(1, 127) = 81.70, *p* < .001, *η*_*p*_^2^ = .391, indicating fear decreased with increased writing repetitions. For expected joy, there was a significant main effect of time, *F*(3, 381) = 5.05, *p* = .002, *η*_*p*_*^2^* = .038, a significant main effect of group, *F*(1, 127) = 20.41, *p* < .001, *η*_*p*_*^2^* = .138, and a significant interaction between time and group, *F*(3, 381) = 3.80, *p* = .010, *η*_*p*_*^2^* = .029. Separate trend analyses showed a linear trend, *F*(1, 61) = 7.20, *p* = .009, *η*_*p*_*^2^* = .106, and a quadratic trend in the control group, *F*(1, 61) = 14.33, *p* < .001, *η*_*p*_*^2^* = .190, indicating that joy decreased with increased writing repetitions in the control group. By contrast, in the expressive writing group, no significant trends were observed, all *p*s > .504, indicating that joy remained stable across writing sessions.

#### Controllability

For expected fear, there was neither a significant main effect of time, Greenhouse–Geisser *F*(2.63, 333.45) = 1.03, *p* = .375, *η*_*p*_*^2^* = .008, nor a significant main effect of group, Greenhouse–Geisser *F*(1, 127) = 1.88, *p* = .173, *η*_*p*_*^2^* = .015, nor a significant interaction between time and group, Greenhouse–Geisser *F*(2.63, 333.45) = 0.54, *p* = .630, *η*_*p*_*^2^* = .004. For expected joy, there was no significant main effect of time, Greenhouse–Geisser *F*(2.78, 353.49) = 0.52, *p* = .658, *η*_*p*_*^2^* = .004, and no significant interaction between time and group, *F*(2.78, 353.49) = 1.87, *p* = .139, *η*_*p*_*^2^* = .015. However, there was a significant main effect of group, Greenhouse–Geisser *F*(1, 127) = 12.28, *p* = .001, *η*_*p*_*^2^* = .088, indicating that, overall, expected joy was higher in the expressive writing group than in the control group. Separate trend analyses showed a linear trend in the control group, *F*(1, 61) = 4.46, *p* = .039, *η*_*p*_*^2^* = .068, indicating that controllability for expected joy decreased with increased writing repetitions in the control group. By contrast, in the expressive writing group, no significant trends were observed, all *p*s > .442, indicating that joy-related controllability remained stable across writing sessions.

#### Emotional burden (fear) and positive motivational activation (joy)

For expected fear, there was a significant main effect of time, Greenhouse–Geisser *F*(2.62, 333.21) = 29.46, *p* < .001, *η*_*p*_*^2^* = .188 and neither a significant main effect of group, Greenhouse–Geisser *F*(1, 127) = 1.18, *p* = .280, *η*_*p*_*^2^* = .009, nor a significant interaction between time and group, Greenhouse–Geisser *F*(2.62, 333.21) = 1.12, *p* = .337, *η*_*p*_*^2^* = .009. Trend analyses showed that there was a linear trend, *F*(1, 127) = 57.02, *p* < .001, *η*_*p*_*^2^* = .310, and a quadratic trend, *F*(1, 127) = 7.46, *p* = .007, *η*_*p*_*^2^* = .056, indicating that the expected emotional burden decreased with an increased number of writing sessions. For expected joy, there was a significant main effect of time, *F*(3, 381) = 4.17, *p* = .006, *η*_*p*_*^2^* = .032, and neither a significant main effect of group, *F*(1, 127) = 2.11, *p* = .149, *η*_*p*_*^2^* = .016, nor a significant interaction between time and group, *F*(3, 381) = 1.06, *p* = .367, *η*_*p*_*^2^* = .008.

#### Expected role in comparison with other expected emotions

For expected fear, there was a significant main effect of time, Greenhouse–Geisser *F*(2.50, 317.71) = 8.75, *p* < .001, *η*_*p*_*^2^* = .064, and neither a significant main effect of group, Greenhouse–Geisser *F*(1, 127) = 0.18, *p* = .673, *η*_*p*_*^2^* = .001, nor a significant interaction between time and group, Greenhouse–Geisser *F*(2.50, 317.71) = 0.71, *p* = .522, *η*_*p*_*^2^* = .006. Trend analyses showed that there was a linear, *F*(1, 127) = 18.23, *p* < .001, *η*_*p*_*^2^* = .126, indicating that the expected role the fear-inducing event will play in future professional life in comparison with other expected emotions decreased with an increased number of writing sessions. For expected joy, there was a significant main effect of time, Greenhouse–Geisser *F*(2.64, 335.67) = 3.37, *η*_*p*_*^2^* = .026, and no significant main effect of group, Greenhouse–Geisser *F*(1, 127) = 1.97, *p* = .163, *η*_*p*_*^2^* = .015. The interaction between time and group was marginally significant, Greenhouse–Geisser *F*(2.64, 335.67) = 2.20, *p* = .096, *η*_*p*_*^2^* = .017. Separate trend analyses showed a linear trend, *F*(1, 66) = 3.46, *p* = .067, *η*_*p*_*^2^* = .050, and a quadratic trend in the expressive writing group, *F*(1, 66) = 7.73, *p* = .007, *η*_*p*_*^2^* = .105, indicating that the expected role of joy increased with increased writing repetitions in the expressive writing group. By contrast, in the control group, no significant trends were observed, all *p*s > .084.

### Motivation to use expressive writing in the future

The motivation to use expressive writing in the future was higher in the expressive writing group, *M* = 3.47, *SD* = 0.91, compared to the control group, *M* = 2.74, *SD* = 0.96, *t*(127) = 4.41, *p* < .001, *d* = 0.78. 71.6% of the participants in the expressive writing group rated their motivation to use expressive writing in the future above the scale midpoint, indicating that the majority of participants found the expressive writing task so useful that they can imagine using it in the future. To provide a more fine-grained picture, in Table 1, means, standard deviations as well as the results of separate *t*-test for comparisons between the expressive writing group and the control group are shown for the four individual items of the scale.

**Table 1:** Motivation to use writing exercises in the future. Means, standard deviations, and results of separate *t*-test for comparisons between the expressive writing group and the control group are shown for the four individual items of the scale (scales ranging from ranging from 1 = no to 5 = yes).

		*Mean*	*SD*	*t*	*p*	*d*
Item 1: Do you think these writing exercises make sense?	Treatment group Control group	3.93 3.19	0.88 0.94	4.58	< .001	0.82
Item 2: Did you find these writing exercises helpful?	Treatment group Control group	3.90 2.95	1.08 1.00	5.16	< .001	0.91
Item 3: Can you imagine using the writing exercises outside the study?	Treatment group Control group	3.25 2.58	1.12 1.21	3.28	.001	0.58
Item 4: Can you imagine continuing using the writing exercises in the long term?	Treatment group Control group	2.79 2.24	1.16 1.18	2.66	.009	0.47

### Change in the view about one’s future professional life

The expressive writing group reported a stronger change in the view about their future professional life as a teacher, *M* = 3.44, *SD* = 0.80, compared to the control group, *M* = 2.70, *SD* = 0.85), *t*(127) = 5.06, *p* < .001, *d* = 0.90. To provide a more fine-grained picture, in Table 2, means, standard deviations as well as the results of separate *t*-test for comparisons between the expressive writing group and the control group are shown for the seven individual items of the scale.

**Table 2:** Change in the view about one’s future professional life. Means, standard deviations, and results of separate *t*-test for comparisons between the expressive writing group and the control group are shown for the seven individual items of the scale (scales ranging from ranging from 1 = no to 5 = yes).

		*Mean*	*SD*	*t*	*p*	*d*
Item 1: Did the writing exercises make you think more about your future professional life as a teacher?	Treatment group Control group	3.87 2.89	0.98 1.34	4.69	< .001	0.84
Item 2: Do you now, after the writing exercises, think differently about your future professional life as a teacher?	Treatment group Control group	2.72 2.34	1.20 1.12	1.84	.067	0.33
Item 3: Do you now, after the writing exercises, feel different about your future everyday working life as a teacher?	Treatment group Control group	2.90 2.37	1.21 1.06	2.61	.010	0.47
Item 4: Did the writing exercises motivate you to prepare yourself more actively for your future professional life as a teacher (e.g., selecting certain courses, etc.)?	Treatment group Control group	3.63 2.90	1.17 1.25	3.40	.001	0.61
Item 5: Do you have the impression that something has changed in you or has already changed as a result of the writing exercises?	Treatment group Control group	3.78 2.85	1.13 1.19	4.53	< .001	0.81
Item 6: Did the writing exercises help you develop different views about your future professional life as a teacher? (e.g., fear)	Treatment group Control group	3.58 2.89	1.06 1.22	3.47	.001	0.61
Item 7: Did the writing exercises help you develop different views about your future professional life as a teacher? (e.g., joy)	Treatment group Control group	3.58 2.65	1.21 1.23	4.36	< .001	0.76

## Discussion

The present findings suggest that expressive writing may be a promising way to promote teacher students’ expected future teaching-related emotions. Writing on three consecutive days for ten minutes each about the future teaching-related event one is most afraid of and the future teaching-related event one is most looking forward to promoted the functionality of expected teaching-related emotions and changed the view about one’s future professional life as a teacher in a functional way.

As hypothesized, regarding positive and negative affect associated with one’s future professional life as a teacher, expressive writing brought about an improvement in that expected global negative affect decreased and expected global positive affect increased with increased writing sessions. Furthermore, as hypothesized, with increased writing sessions, the expected emotional burden due to the fear-inducing event one had written about decreased, and the expected emotional reward due to the joy-inducing event one had written about increased. Beyond these emotional effects, after the expressive writing sessions, as hypothesized, teacher students in the expressive writing group compared to the control group reported a more active personal involvement with their future professional life and a stronger motivation to use expressive writing in the future. In addition, teacher students in the expressive writing condition reported a stronger change in their view about their future professional life as a teacher.

These findings support previous findings showing that expressive writing is a helpful technique to promote the functional reprocessing of and coping with past negative events and to increase the benefits from past positive events [for reviews, see, e.g., 3; 25]. Going beyond previous findings, our study demonstrates that expressive writing cannot only promote the functional dealing with emotions elicited by past events but also the functionality of expected emotions elicited by future events. In particular, since teacher students reported additionally a change in their view about and a more active involvement with their future professional life, the present findings demonstrate that expressive writing is not only helpful regarding expected emotions but also regarding cognitions about one’s future professional life, which is in line with previous findings indicating that writing can promote reflection [32].

While the present findings indicate that not only writing about past emotional experiences but also writing about expected future emotional experiences can be beneficial for psychological well-being, there are a number of differences between past emotional experiences and expected future emotional experiences such as, for instance, differences in the intensity of elicited emotions or differences in the ability to imagine past versus future events, which could make a difference in terms of the effects of expressive writing. Examining potential differences in the effects of expressive when writing about past versus expected future experiences may be an interesting avenue for future research.

An interesting finding of the present study beyond the effects of expressive writing is that also writing about an unrelated event after having imagined future emotional events can have positive effects on expected future emotions. Especially with regard to future fear-inducing events, writing about a walk in a park or a forest decreased the expected global negative affect and the expected emotional burden due to future fear-inducing events to the same extent than expressive writing. Such a finding suggests that imagining and writing down future fear-inducing events once and writing afterwards about unrelated topics may be sufficient to promote functional expected emotions, a finding which supports evidence showing the existence of nonconscious emotional reappraisal processes [39]. Since numerous studies have shown that walking in nature or viewing pictures of nature can improve psychological functioning [40], the observed improvement in expected emotions after writing about a walk in a park or a forest may also be related to such nature-induced effects, which may be an interesting question for future research. Nevertheless, regarding teacher training, the use of expressive writing is favorable over writing about unrelated topics due to the additional beneficial effects of expressive writing on the view of and the involvement with the future professional life as a teacher, and the increased motivation to use expressive writing in the long term.

When comparing the effects of expressive writing for the fear-inducing and the joy-inducing events, the beneficial effect of expressive writing on expected emotions were stronger for the fear-inducing than for the joy-inducing events. However, rather than reflecting the possibility that expressive writing may be more effective for fear-inducing than for joy-inducing events, it may alternatively be that this finding reflects a ceiling effect in the joy condition. Expected joy regarding the future teaching-related event one is most looking forward to was already largely at ceiling at baseline (e.g., mean intensity of joy at baseline: 6.55 on a scale of 1 = very low to 7 = very high) so that few room for improvement was left. By contrast, since improvement in the case of fear means a decrease in intensity, there was much more room for improvement regarding the future teaching-related event one is most afraid of (mean intensity of fear at baseline: 4.74 on a scale of 1 = very low to 7 = very high). To clarify whether expressive writing may be similar or even more effective for joy-inducing than for fear-inducing events, further research is necessary where joyful events are used that are less emotionally intense.

A final interesting observation is that the participants showed a more active involvement with their later professional life and a higher motivation to use expressive writing in the long term despite writing about fear-inducing future teaching-related events. This finding is important because it shows that expressively writing about future fear-inducing events does not induce avoidance behavior but increase the willingness to invest resources to solve the underlying problem. However, since the participants in the present study wrote both about joy-inducing and fear-inducing topics, it may be that concurrently writing about positive topis of future life may be an important precondition that writing about negative topics does not lead to avoidance behavior, a question that requires further research.

In conclusion, the present study provides evidence that expressive writing may be a very promising way to promote teacher students’ expected teaching-related emotions. Regarding teacher training, beyond the beneficial effects on expected emotions and on the view of and involvement with later professional life, it is noteworthy that expressive writing is a very feasible technique which only takes little time and can easily be implemented in an online environment without the need to provide individual support. Thus, expressive writing may be an effective and feasible tool to help teacher students to become better teachers.

## References

[1] Pennebaker JW, Beall SK. Confronting a traumatic event: Toward an understanding of inhibition and disease. J Abnorm Psychol. 1986;95(3):274–281. doi: 10.1037//0021-843x.95.3.274 3745650

[2] Pennebaker JW, Kiecolt-Glaser JK, Glaser R. Disclosure of traumas and immune function: health implications for psychotherapy. J Consult Clin Psychol. 1988;56(2):239–245. doi: 10.1037//0022-006x.56.2.239 3372832

[3] Pennebaker JW, Chung CK. Expressive writing and its links to mental and physical health. In Friedman HS, editor. Oxford handbook of health psychology. Oxford: Oxford; 2011.

[4] Pennebaker JW. Writing about emotional experiences as a therapeutic process. Psychol Sci. 1997;8(3):162–166. doi: 10.1111/j.1467-9280.1997.tb00403.x

[5] Krpan K. M., Kross E., Berman M. G., Deldin P. J., Askren M. K., & Jonides J. An everyday activity as a treatment for depression: The benefits of expressive writing for people diagnosed with major depressive disorder. Journal of Affective Disorders 2013; 150(3), 1148–1151. doi: 10.1016/j.jad.2013.05.065 23790815 PMC3759583

[6] Gortner EM, Rude SS, Pennebaker JW. Benefits of Expressive Writing in Lowering Rumination and Depressive Symptoms[J]. Behavior Therapy. 2006;37(3):292–303. doi: 10.1016/j.beth.2006.01.004 16942980

[7] McGuire K. M. B., Greenberg M. A., and Gevirtz R., “Autonomic effects of expressive writing in individuals with elevated blood pressure,” Journal of Health Psychology 2005, vol. 10, no. 2, p. 197–209. doi: 10.1177/1359105305049767 15723890

[8] Pennebaker JW, Francis ME. Cognitive, emotional, and language processes in disclosure. Cogn Emot. 1996;10(6):601–626. doi: 10.1080/026999396380079

[9] Francis ME, Pennebaker JW. Putting stress into words: The impact of writing on physiological, absentee, and self-reported emotional well-being measures. Am J Health Promot. 1992;6(4):280–287. doi: 10.4278/0890-1171-6.4.280 10146806

[10] Yang Z., Tang X., Duan W., & Zhang Y. Expressive writing promotes self‐reported physical, social and psychological health among Chinese undergraduates. International Journal of Psychology 2015; 50(2), 128–134. doi: 10.1002/ijop.12081 24903848

[11] Park D., Ramirez G., & Beilock S. L. The role of expressive writing in math anxiety. *Journal of Experimental Psychology*: *Applied* 2014; 20(2), 103–111. doi: 10.1037/xap0000013 24708352

[12] Shen L., Yang L., Zhang J., & Zhang M. Benefits of expressive writing in reducing test anxiety: A randomized controlled trial in Chinese samples. Plos one 2018; 13(2), e0191779. doi: 10.1371/journal.pone.0191779 29401473 PMC5798770

[13] Ashley Laura, O’Connor & Fiona Jones Daryl B. A randomized trial of written emotional disclosure interventions in school teachers: Controlling for positive expectancies and effects on health and job satisfaction, Psychology, Health & Medicine 2013; 18:5, 588–600, doi: 10.1080/13548506.2012.756536 23323573

[14] Burton CM, King LA. The health benefits of writing about intensely positive experiences. J Res Pers. 2004;38(2):150–163. doi: 10.1016/S0092-6566(03)00058-8

[15] Allen SF, Wetherell MA, Smith MA. Online writing about positive life experiences reduces depression and perceived stress reactivity in socially inhibited individuals. Psychiatry Res. 2020;284:Article 112697. doi: 10.1016/j.psychres.2019.112697 31791707

[16] King L. A., “The health benefits of writing about life goals,” Personality and Social Psychology Bulletin 2001; vol. 27, no. 7, pp. 798–807.

[17] Smith MA, Thompson A, Hall LJ, Allen SF, Wetherell MA. The physical and psychological health benefits of positive emotional writing: Investigating the moderating role of Type D (distressed) personality. Br J Health Psychol. 2018;23(4):857–871. doi: 10.1111/bjhp.12320 29862618 PMC6174944

[18] King L.A., Miner K.N. Writing about the perceived benefits of traumatic events: implications for physical health. Personality & Social Psychology Bulletin 2000; 26 (2), 220–230.

[19] Round E., Wetherell M., Elsey V., & Smith M. A. Positive expressive writing as a tool for alleviating burnout and enhancing wellbeing in teachers and other full-time workers. 2020.

[20] Jones B. K., & Destin M. Effects of positive versus negative expressive writing exercises on adolescent academic achievement. Journal of Applied Social Psychology 2021; 51(6), 549–559. doi: 10.1111/jasp.12756

[21] Wong C. S., Chua M. J., & Prihadi K. D. Reducing depressive symptoms and increasing positive feelings with expressive writing. International Journal of Public Health 2021; 10(2), 433–444. doi: 10.11591/ijphs.v10i2.20797

[22] Kloss J. D. and Lisman S. A., “An exposure‐based examination of the effects of written emotional disclosure,” British Journal of Health Psychology 2002; vol. 7, no. 1, pp. 31–46. doi: 10.1348/135910702169349 14596716

[23] Smyth JM. Written emotional expression: effect sizes, outcome types, and moderating variables. J Consult Clin Psychol. 1998;66(1):174–184. doi: 10.1037//0022-006x.66.1.174 9489272

[24] Frisina PG, Borod JC, Lepore SJ. A meta-analysis of the effects of written emotional disclosure on the health outcomes of clinical populations. J Nerv Ment Dis. 2004;192(9):629–634. doi: 10.1097/01.nmd.0000138317.30764.63 15348980

[25] Frattaroli J. Experimental disclosure and its moderators: a meta-analysis. Psychol Bull. 2006;132(6):823–865. doi: 10.1037/0033-2909.132.6.823 17073523

[26] Van Emmerik AA, Reijntjes A, Kamphuis JH. Writing therapy for posttraumatic stress: a meta-analysis. Psychother Psychosom. 2013;82(2):82–88. doi: 10.1159/000343131 23295550

[27] Meads C, Nouwen A. Does emotional disclosure have any effects? A systematic review of the literature with meta-analyses. Int J Technol Assess Health Care. 2005;21(2):153–164. doi: 10.1017/S026646230505021X 15921054

[28] Harris AH. Does expressive writing reduce health care utilization? A meta-analysis of randomized trials. J Consult Clin Psychol. 2006;74(2):243–252. doi: 10.1037/0022-006X.74.2.243 16649869

[29] Mogk C, Otte S, Reinhold-Hurley B, Kröner-Herwig B. Health effects of expressive writing on stressful or traumatic experiences-a meta-analysis. GMS Psycho-Social Medicine. 2006;3:Article 19742069. . 19742069 PMC2736499

[30] Travagin G, Margola D, Revenson TA. How effective are expressive writing interventions for adolescents? A meta-analytic review. Clin Psychol Rev. 2015;36:42–55. doi: 10.1016/j.cpr.2015.01.003 25656314

[31] Reinhold M, Bürkner PC, Holling H. Effects of expressive writing on depressive symptoms—A meta‐analysis. Clin Psychol (New York). 2018;25(1):Article e12224. doi: 10.1111/cpsp.12224

[32] Bain JD, Ballantyne R, Packer J, Mills C. Using journal writing to enhance student teachers’ reflectivity during field experience placements. Teachers and Teaching. 1999;5(1):51–73. doi: 10.1080/1354060990050104

[33] O’Connor RC, Williams JMG. The relationship between positive future thinking, brooding, defeat and entrapment. Personal Individ Differ 2014;70: 29–34. doi: 10.1016/j.paid.2014.06.016

[34] Nolen-Hoeksema S. The role of rumination in depressive disorders and mixed anxiety/depressive symptoms. J Abnorm Psychol. 2000;109(3):504–511. doi: 10.1037/0021-843X.109.3.504 11016119

[35] Rivkin ID, Taylor SE. The effects of mental simulation on coping with controllable stressful events. Pers Soc Psychol Bull. 1999;25(12):1451–1462. doi: 10.1177/2F01461672992510002

[36] Leiner DJ. SoSci survey. Version 3.2.12 [software]. 2020 Oct 23 [2021 Sep 24]. Available from: https://www.soscisurvey.de

[37] Breyer B, Bluemke M. [German version of the Positive and Negative Affect Schedule PANAS (GESIS Panel)]. Zusammenstellung sozialwissenschaftlicher Items und Skalen (ZIS). 2016;10:1–20. German. doi: 10.6102/zis242

[38] Watson D, Clark LA, Tellegen A. Development and validation of brief measures of positive and negative affect: the PANAS scales. J Pers Soc Psychol. 1988;54(6):1063–1070. doi: 10.1037//0022-3514.54.6.1063 3397865

[39] Yuan J, Ding N, Liu Y, Yang J. Unconscious emotion regulation: Nonconscious reappraisal decreases emotion-related physiological reactivity during frustration. Cogn Emot. 2015;29(6):1042–53. doi: 10.1080/02699931.2014.965663 25297822

[40] Berman MG, Jonides J, Kaplan S. The Cognitive Benefits of Interacting With Nature. Psychological Science. 2008;19(12):1207–1212. doi: 10.1111/j.1467-9280.2008.02225.x 19121124

